# Cushing´s syndrome due to bilateral adrenal cortical disease: Bilateral macronodular adrenal cortical disease and bilateral micronodular adrenal cortical disease

**DOI:** 10.3389/fendo.2022.913253

**Published:** 2022-08-05

**Authors:** Marta Araujo-Castro, Mónica Marazuela

**Affiliations:** ^1^ Endocrinology & Nutrition Department, Ramón y Cajal University Hospital, Madrid, Spain; ^2^ Instituto Ramón y Cajal de Investigación Sanitaria IRYCIS, Madrid, Spain; ^3^ Departament of Medicine, Alcalá University, Madrid, Spain; ^4^ Endocrinology & Nutrition Department, La Princesa University Hospital, Madrid, Spain

**Keywords:** Cushing´s syndrome, bilateral macronodular adrenal cortical disease, bilateral micronodular adrenal cortical disease, primary pigmented nodular adrenocortical disease, adrenalectomy

## Abstract

Cushing´s syndrome (CS) secondary to bilateral adrenal cortical disease may be caused by bilateral macronodular adrenal cortical disease (BMACD) or by bilateral micronodular adrenal cortical disease (miBACD). The size of adrenal nodules is a key factor for the differentiation between these two entities (>1cm, BMACD and <1cm; miBACD). BMACD can be associated with overt CS, but more commonly it presents with autonomous cortisol secretion (ACS). Surgical treatment of BMACD presenting with CS or with ACS and associated cardiometabolic comorbidities should be the resection of the largest adrenal gland, since it leads to hypercortisolism remission in up to 95% of the cases. Medical treatment focused on the blockade of aberrant receptors may lead to hypercortisolism control, although cortisol response is frequently transient. miBACD is mainly divided in primary pigmented nodular adrenocortical disease (PPNAD) and isolated micronodular adrenocortical disease (i-MAD). miBACD can present at an early age, representing one of the main causes of CS at a young age. The high-dose dexamethasone suppression test can be useful in identifying a paradoxical increase in 24h-urinary free cortisol, that is a quite specific in PPNAD. Bilateral adrenalectomy is generally the treatment of choice in patients with overt CS in miBACD, but unilateral adrenalectomy could be considered in cases with asymmetric disease and mild hypercortisolism. This article will discuss the clinical presentation, genetic background, hormonal and imaging features and treatment of the main causes of primary bilateral adrenal hyperplasia associated with hypercortisolism.

## 1 Introduction

Twenty percent of cases of Cushing’s syndrome (CS) are of adrenal origin (1). Although most of adrenal CS are secondary to benign cortisol-secreting adenomas, up to 10% of cases are secondary to primary adrenal hyperplasia or bilateral adrenal cortical disease (1). The new WHO classification of adrenal cortical proliferations refined classification of adrenal cortical nodular disease which now includes (a) sporadic nodular adrenocortical disease, (b) bilateral micronodular adrenal cortical disease (miBACD), and (c) bilateral macronodular adrenal cortical disease (BMACD). This new classification no longer endorses the use of “nodular adrenal cortical hyperplasia” for incidentally discovered sporadic non-functional adrenal cortical nodules. This is based on the recognition of the clonal/neoplastic nature of incidentally discovered non-functional subcentimeter benign adrenal cortical nodules (2). The distinction between these diagnostic categories reflects diverse clinical manifestations (2).

Regarding the different types of bilateral adrenal cortical disease, we differentiate between cases of primary bilateral adrenal cortical disease, presenting with low and high ACTH levels respectively. BMACD and miBACD are included in the low ACTH group. Cushing’s disease and ectopic CS that are mediated by the stimulatory effects of ACTH have high ACTH levels and are included in the group of diffuse bilateral cortical hyperplasia. The reported frequency of adrenal hyperplasia and adrenal nodules in Cushing´s disease ranges between 25% and 50% (3, 4). In glucocorticoid resistance syndrome, the lack of negative feedback by cortisol on the hypothalamic-pituitary-adrenal axis leads to a chronic elevation of ACTH (5). Congenital adrenal hyperplasia (CAH) due to enzyme defects within the adrenal cortex leads to elevated ACTH and adrenal hyperplasia but does not cause hypercortisolism (6) **(** **Table 1** **).**

**Table 1:** Differential diagnosis of bilateral adrenal cortical disease (6).

Type of adrenal hyperplasia	ACTH levels	Biochemical data of hypercortisolism	Specific clinical data of hypercortisolism
**CAUSES OF BILATERAL ADRENAL CORTICAL DISEASE**			
**BMACD**	Low/Low-normal	Present	Usually absent (ACS)
**miBACD** •**PPNAD** •**i-MAD**	Low	Present. Paradoxical increase in UFC in the HDDT in PPNAD	Present (except in cases of ACS)
**CAUSES OF DIFFUSE BILATERAL CORTICAL HYPERPLASIA**			
**Cushing´s disease**	High	Present	Present
**Ectopic Cushing´s syndrome**	High/Very high	Present	Present (except in very aggressive ECS)
**Glucocorticoid resistance syndrome**	High	Present	Absent
**CAH**	High	Absent	Absent

ACS, autonomous cortisol secretion; ECS, ectopic Cushing´s syndrome; CAH, congenital adrenal hyperplasia; HDDST, high-doses dexamethasone suppression test; I-MAD, isolated micronodular adrenocortical disease; PBMAH, primary bilateral macronodular adrenal hyperplasia; miBAH, micronodular bilateral adrenal hyperplasia; PPNAD, primary pigmented nodular adrenocortical disease; UFC, urinary free cortisol.

For the differentiation between the main causes of primary bilateral adrenal cortical disease associated with hypercortisolism, the key factor is the size of the adrenal nodules (6). If they are larger than 1 cm, we would generally be dealing with a case of BMACD, and if they are smaller than 1 cm, it would be a case of miBACD. On the other hand, both conditions are frequently genetically determined, but it is more common in primary pigmented nodular adrenocortical disease (PPNAD). In this last entity, up to 80% of cases are genetic, compared to 25% of the cases of BMACD. The different variants of primary adrenal cortical disease can present with overt CS or only with autonomous cortisol secretion (ACS) (biochemical evidence of hypercortisolism without specific clinical features of CS such as myopathy, ecchymosis, or skin atrophy) (7). BMACD and miBACD do not constitute different stages of the same disease, since they are different entities with different genetic causes, mechanisms, biochemical profiles, and clinical implications (8).

## 2 Bilateral macronodular adrenal cortical disease (BMACD)

### 2.1 Definition

The most accepted definition for BMACD is the existence of bilateral adrenal hyperplasia associated with one or more adrenal nodules larger than 1 cm in each adrenal gland (9). Previously, it was considered that there should be atrophy of the adrenal internodular cortex, but later on it was described that internodular atrophy was not always present. Since then, two histological forms of BMACD have been differentiated: BMACD type 1, which presents with atrophic internodular cortex, and BMACD type 2, which exhibits both nodular and internodular tissue hyperplasia (9). Despite this definition, BMACD is quite a heterogeneous condition, since the size of the nodules can be highly variable, from 1 to 5 cm, and the degree of hypercortisolism can vary from ACS to overt CS. Even then, the asynchronous involvement of only one adrenal gland can also occur in BMACD (10). Cases of mineralocorticoid excess (11), combined with gluco-mineralocorticoid excess (11), cortisol-estrone secretion with expression of P450 aromatase (CYP19A1) (12) or DHEA and DHEAS secretion have also been described.

### 2.2 Pathophysiology

**Figure 1:**
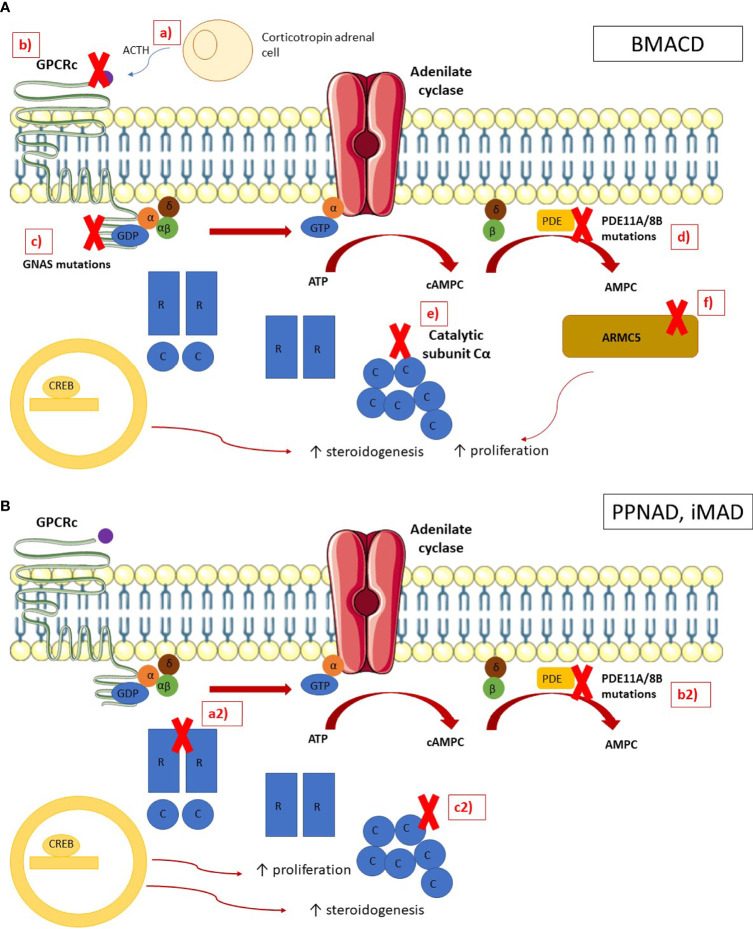
Pathophysiology of bilateral macronodular adrenal cortical disease and bilateral micronodular adrenal cortical disease In normal adrenocortical cells, the MC2R receptor is activated by ACTH, leading to the activation of the G subunits of the latter activates the adenylate cyclase, which converts the ATP in cAMP. The phosphodiesterases (PDE) inactivates cAMP in AMP. cAMP is binding by the regulatory (R) subunits of the PKA, leading to the release of the catalytic (C) subunits. The catalytic subunits phosphorylate their targets, including the cAMP Response Element-Binding protein (CREB), which activates genes involved in steroidogenesis. ARMC5 blocks the cell cycle in G1 phase and induces apoptosis. **(A)** In BMACD, the PKA pathway is activated by **a** ACTH locally produced by clusters of corticotropin adrenal cells, **b** mutations in the gene coding for MC2R, **c** mutations in gene GNAS, **d** mutations in phosphodiesterase genes, **e** duplication of the catalytic subunit C, and **f** ARMC5 mutations, which lead to the activation of the cell cycle and the loss of apoptosis. **(B)** In PPNAD and iMAD, the PKA pathway is activated by **(a2)** mutations in the regulatory subunit R of PKA and **(b2)** mutations in phosphodiesterases genes, and **(c2)** duplication of the catalytic subunit.

The pathophysiology of BMACD is complex as the adrenal process of growth and hypersecretion could be explained through different mechanisms. Hormonal hypersecretion and adrenal nodule growth is related to the activation of the cAMP/protein kinase A (PKA) pathway. Theoretically, disruptions in each step of this pathway may be involved in the pathogenesis, such as activating mutations in melanocortin 2 receptor (MC2R), mutations in the alpha subunit of stimulatory G proteins (GNAS) or a decreased activity of phosphodiesterases (13) (**Figure 1**).

Additionally, in a significant proportion of cases, between 77% and 87% (14), an activation of the cAMP/PKA pathway through stimulation by ligands other than ACTH of aberrantly expressed G protein-coupled receptors has been identified. There are two types of aberrant receptors, those that are ectopically expressed and those that are eutopic but are overexpressed (15). Among those with ectopic expression, there are receptors for gastric inhibitory polypeptide (GIP), catecholamines, vasopressin (V2-V3), serotonin (5-HT7), and angiotensin (AT1). Among those that are eutopic but overexpressed, are vasopressin receptor type 1 (V1), LH/hCG-R, serotonin type 4 (5-HT4) and leptin receptors. Up to 50% of patients respond to four or more stimuli (14). The most frequently detected are vasopressin type 1 (V1) and serotonin type 4 (5-HT4) (14). The overall prevalence and magnitude of aberrant cortisol regulation are similar in patients with overt CS and ACS (16) (**Table 2**). The presence of aberrant receptors is not specific to BMACD, as they have been described in 30% of bilateral and unilateral adrenal hyperplasia with associated hypercortisolism (18).

**Table 2:** Aberrant expression of G-coupled protein receptor in BMACD, and their screening protocols and specific medical therapy (17).

Aberrant receptor	Protocol used for its detection	Medical treatment
GIP receptor	Mixed meal	Octreotide, pasireotide
Vasopressin 1,2 and 3 receptors	Desmopressin administration	Vasopressin receptor antagonist
β-adrenergic receptor	Insulin hypoglycemia	B-blockers (propranolol)
5-HT4 receptor	Metoclopramide administration	5-HT4 receptor antagonist
LH-hCG receptor	LH or hCG infusion	Long-acting GnRH agonist
AT1-receptor	Upright posture test	AT-1 receptor antagonist
Glucagon receptor	Intravenous glucagon infusion	No specific treatment
Prolactin receptor	TRH administration	No specific treatment

GIP, gastric inhibitory polypeptide; AT1, angiotensin II type 1.

Another mechanism involved in the pathogenesis of BMACD is the local production of ACTH, by the steroidogenic cells themselves (19). Aberrant receptors that regulate ACTH secretion through a paracrine/autocrine mechanism, which would stimulate the production of glucocorticoids and adrenal nodular growth, have been detected in patients with BMACD. Due to the discovery of this mechanism, the term ACTH-independent macronodular adrenal hyperplasia is no longer used. Other pathways, such as Wnt/b-catenin activation, have also been implicated in the stimulation of adrenal node growth (17, 20).

### 2.3 Genetic background

BMACD may very rarely be due to genetic alterations in MC2R (18p11.21) (21), protein kinase cAMP-activated catalytic subunit alpha (PRKACA) (22) and phosphodiesterase isoform 11A (PDE11A) (23). In addition, BMACD can rarely be part of familial syndromes such as McCune Albright syndrome, multiple endocrine neoplasia type 1 (MEN1) syndrome (11q13.1), familial adenomatous polyposis coli (5q21-22) or hereditary leiomyomatosis and renal cell carcinoma (HLRCC) (1p42.3e43). It is estimated that up to 30% of MEN1 patients have adrenal involvement and in 13% of them it is bilateral disease, although the cases of BMACD described in MEN1 are scarce (24). Familial adenomatous polyposis coli or Gardner’s syndrome is associated with adrenal disease in almost a quarter of the patients, and in almost another quarter there is bilateral involvement (25). Familial leiomyomatosis syndrome has adrenal involvement in 8% of the cases, this being bilateral in 15% of them (26). However, also in these two conditions cases of BMACD are anecdotal. The familial condition most associated with BMACD is the McCune Albright syndrome due to germline mutations in the GNAS gene (20q13.32). This disorder presents with BMACD in 7% of patients, and it usually manifests in the first years of life (13). The syndrome presents with a classic triad of polyostotic fibrous dysplasia, ‘cafe´ au latte´’ skin spots, as well as isosexual, gonadotropin-independent precocious puberty (27).

Although BMACD was initially considered to be sporadic, several familial cases with an autosomal dominant pattern of transmission due to germline mutations in the Armadillo repeat-containing protein 5 (ARMC5) gene (16p11.2) have been described in the last decades. Mutations in ARMC5 reduce the ability to secrete steroids by the cells, which is consistent with what has already been described regarding the presence of ineffective steroidogenesis in patients with BMACD, for which reason 24h-urinary free cortisol (UFC) levels are lower than expected, and cortisol precursors are increased (17). The prevalence of germline pathogenic ARMC5 mutations is estimated to be 20%-30% in various cohorts of nonfamilial BMACD cases (9, 21). In the Assie et al. series that included 33 patients with BMACD and overt CS, germline mutations in ARMC5 were detected in 55% of cases (28). The presence of mutations is significantly more frequent in cases with overt CS (28-55%) than in cases with ACS, where the prevalence is around 10% (17). It also seems that the presence of the mutation is more frequent in those patients with larger adrenal glands and with multiple macronodules, and it is also associated with more severe phenotypes, with a higher prevalence of overt CS (29). The search for ARMC5 or other responsible genes should allow familial screening, permitting the identification of at-risk individuals who would probably benefit from further evaluations (13).

Regarding germline mutations in ARMC5, it has been suggested that it could play a pathogenic role in the development of other tumors such as intracranial meningiomas. The coexistence of BMACD and intracranial meningioma was described for the first time in 2005 in two siblings with BMACD and meningioma (30). Subsequently, meningiomas were reported in 3 members of a Brazilian family of 7 members with BMACD in 2014, suggesting the possibility of a pathogenic role of ARMC5 (10). More recently, Elbet et al. (31) performed gene sequencing both at the germinal level and in tumor samples, finding that in one of the patients with BMACD and meningioma had an ARMC5 somatic mutation, so they concluded that the biallelic inactivation of ARMC5 could be a cause of intracranial meningioma. Nonetheless, further studies are needed to confirm the potential involvement of ARMC5 mutations with the co-occurrence of other extra-adrenal tumors.

Moreover, recently, a new germline truncating variant of KDM1A has been described as a cause of hereditary food-dependent CS in patients with BMACD (32). KDM1A inactivation explains about 90% of food-depended CS cases of BMACD (33). It has been described that those patients with KDM1A mutations have a higher risk of developing monoclonal gammopathies, so it is recommended to perform a serum protein electrophoresis study in all patients with KDM1A mutations (32).

### 2.4 Epidemiology and clinical presentation

BMACD is a rare and insidious cause of CS, and accounts for less than 2% of all cases of endogenous CS (34). Hypercortisolism usually presents an insidious course, which progresses slowly over the years, so it may take several years until the diagnosis. In fact, many patients are diagnosed incidentally when performing imaging tests for other reasons unrelated to suspected adrenal disease (35). Nevertheless, it seems to be some difference in the severity of the hypercortisolism according to age, being more frequent the cases of overt CS in young patients. These differences may be related to the fact that a younger age at presentation is more commonly associated with germline ARMC5 mutations (17). Once considered a rare disease, BMACD is now encountered with increasing frequency, mainly due to the increased detection of adrenal incidentalomas (36).

In sporadic cases, there is a higher prevalence in women than in men, whereas in hereditary cases BMACD affects men and women equally (37). In patients with McCune-Albright syndrome, BMACD is usually diagnosed between 5-10 years of age; while in sporadic cases it is usually between the fifth and sixth decades of life (37).

In patients with a response to GIP, the presence of fasting hypocortisolism has been described, especially at 8 am (38). In patients who have LH/hCG receptors, there have been reports of CS manifesting either in pregnancy or menopause, with the rise in LH/FSH (39).

### 2.5 Radiological diagnosis

**Figure 2:**
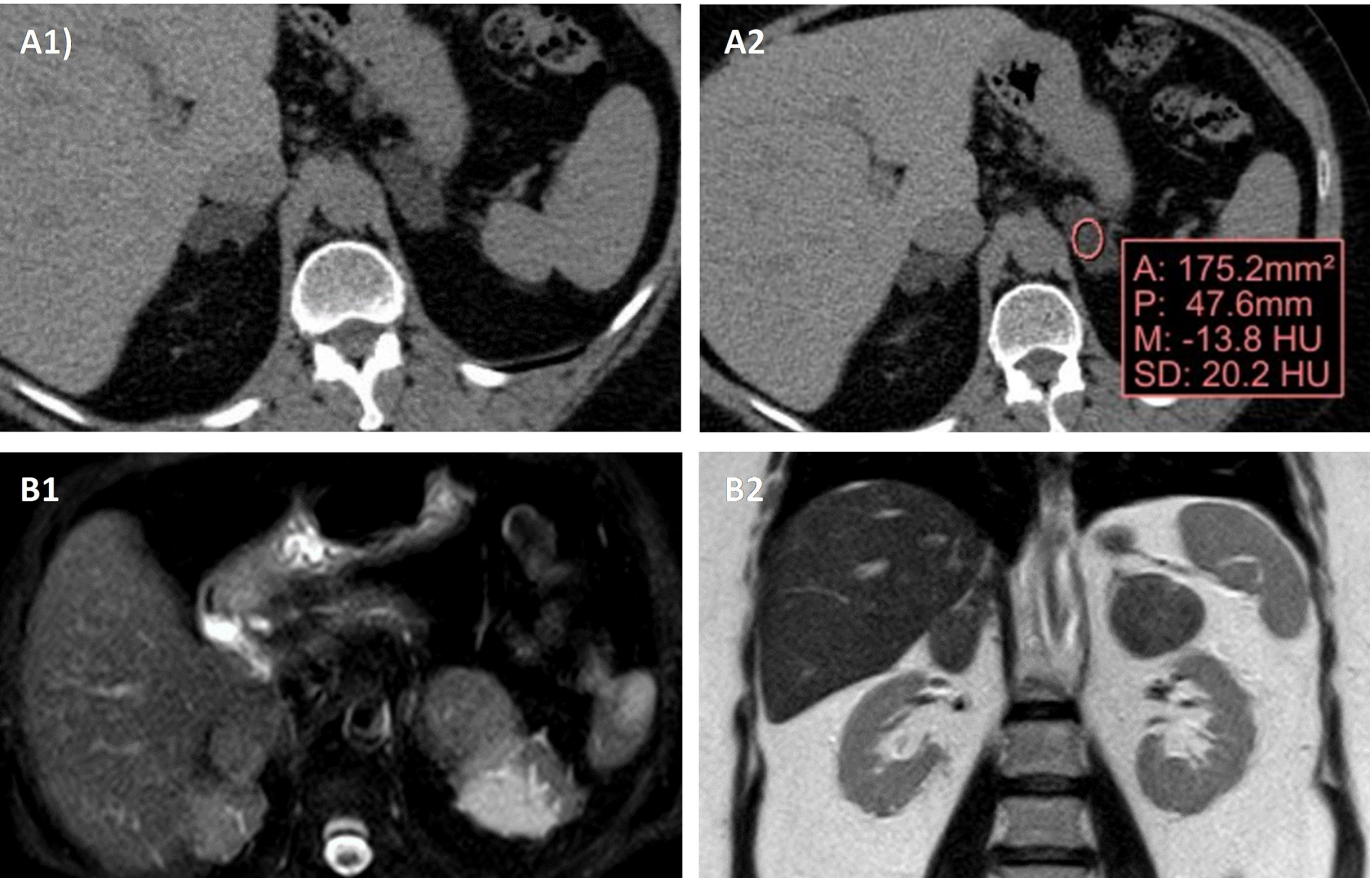
Radiological appearance of BMACD in the unenhanced CT and MRI. Unenhanced CT shows bilateral adrenal hyperplasia secondary to the existence of several well-defined, confluent nodular lesions **(A1)**, with negative density values in the non-contrast CT, compatible with lipid-rich adenomas **(A2)**. In MRI, axial **(B1)** and coronal **(B2)** sections, there are a right nodular conglomerate of 46x19x29 mm, and in the left adrenal gland 2 nodules of 34x17 mm and 26x21 mm, compatible with adenomas.

For the radiological diagnosis of BMACD, as in the evaluation of adrenal incidentalomas, unenhanced CT is usually considered the imaging technique of choice (9). The radiological presentation is variable, from obvious cases with multiple macronodules in each adrenal gland, to cases that only have one single macronodule in each gland. The differential diagnosis should be made mainly with other bilateral adrenal lesions (9) ***(*** **Figure 2** ***).*** It is important above all to rule out adrenal metastases, infiltrative diseases, tuberculosis, fungal infections, lymphoma, myelolipomas, bilateral adrenal carcinoma, and bilateral pheochromocytoma, although these last two conditions usually present as unilateral forms (40, 41). However, it must be emphasized that in all these conditions with the exception of bilateral adrenal carcinoma, there is no overproduction of cortisol, being more common the presence of hypoadrenalism (41).

In the evaluation of adrenal lesions, Hounsfield units (HU) measurement <10 on unenhanced CT usually indicates a lipid-rich lesion, that can be diagnosed as benign (41). In this way, although in general the presence of an adrenal lesion with >10 HU should alert us and force us to rule out malignant adrenal disease and pheochromocytoma, cases of adrenal lesions with 10 HU have been described in patients with BMACD (42, 43). One study reported that approximately 61% of the adrenal macronodules manifested a density >10 HU and 30% manifested a density >20 HU (29). On magnetic resonance imaging (MRI) glands are usually hypointense on T1-weighted images and hyperintense on T2-weighted images (42).

There are some cases of BMACD where [18F]-2-fluoro-deoxy-D-glucose FDG positron emission tomography/computed tomography (PET/CT) imaging showed a higher uptake in the adrenals than in the liver, with maximum standardized uptake value (SUV max) reaching 5.5 despite the benign nature of the disease (44). In these patients, FDG-PET uptake may have a functional correlate. Supporting this theory, Akkus G. et al.(45) found that SUVmax values on the FGD-PET were significantly lower in patients with non-functional adrenal masses (SUVmax 3.2) than in functional adrenal masses (SUVmax 10.1). Moreover, cortisol-secreting adrenal masses showed the highest SUVmax value (10.1) among the secreting tumors.

Adrenal biopsy is not usually indicated in these patients, since it has limited reliability for the differential diagnosis between adrenocortical adenoma and carcinoma and can lead to tumor spread. It is usually reserved to patients with an active or previously diagnosed extra-adrenal malignancy (46).

### 2.6 Biochemical diagnosis

The biochemical diagnosis will depend on whether the patient has clinical features of CS, in which case we will carry out a study aimed to diagnose hypercortisolism (47), or if it is diagnosed in the context of bilateral adrenal incidentalomas. In this second case, in addition to ruling out malignancy through imaging tests, it is recommended to screen for pheochromocytoma and hypercortisolism. Moreover, in patients with hypertension and/or hypokalemia, primary hyperaldosteronism should also be discarded. In addition, since these are bilateral lesions, adrenal insufficiency should be evaluated if there is suspicion of malignant or infiltrative disease, and of CAH (46, 48).

Regarding the screening of hypercortisolism, the test recommended by the different guidelines to carry out the screening is the low-dose dexamethasone suppression test (DST) (46, 49). ACS is defined as a cortisol post-DST > 5 µg/dL without specific features of CS, and possible ACS when values are between 1.9-5 µg/dl (49). In these cases, with abnormal results in the DST, other studies to assess the degree of hypercortisolism such as UFC and midnight cortisol should be considered (9). Nevertheless, there is no general agreement on the definition of ACS as no test or combination of tests have been demonstrated to be fully reliable in diagnosing subtle cortisol excess (7). However, in general, the important point is that the insufficient cortisol suppression after DST seems to be associated with comorbidities (7, 46, 49). Moreover, ACTH-dependence should be ruled out by the determination of plasma ACTH levels. However, ACTH levels are not always suppressed, especially in cases with ACS or possible ACS (7). Plasma ACTH will gradually become suppressed as cortisol hypersecretion progresses. The presence of non-suppressed ACTH levels, even high-normal ACTH levels in some cases of BMACD may be explained by the presence of local production of ACTH by the steroidogenic cells themselves (19). Supporting this finding, in our recently published series of BMACD, mean ACTH levels were of 23.6 ± 48.57 pg/mL (36). Other authors reported suppressed ACTH levels only in 30% of the cases (48). The dexamethasone-CRH test is not useful for the differential diagnosis of ACTH dependence since it can cause false positives in a high percentage of bilateral adrenal lesions (~40% of the cases) (50). Patients with PBMAH had a profile of ineffective steroidogenesis that resembles that of adrenocortical carcinoma, with elevation of DHEA-S and 3β-OH-Δ5 metabolites of cortisol (9)

It is also important to keep in mind that these patients may present elevations of 17OH-progesterone, and even response to the ACTH test for 17OH-progesterone. The key factor to differentiate this condition from CAH is that in BMACD there is associated hypercortisolism and usually low ACTH levels while CAH is a condition that often causes hypoadrenalism and increased ACTH concentrations (14).

### 2.7 Study of aberrant receptors

**Figure 3:**
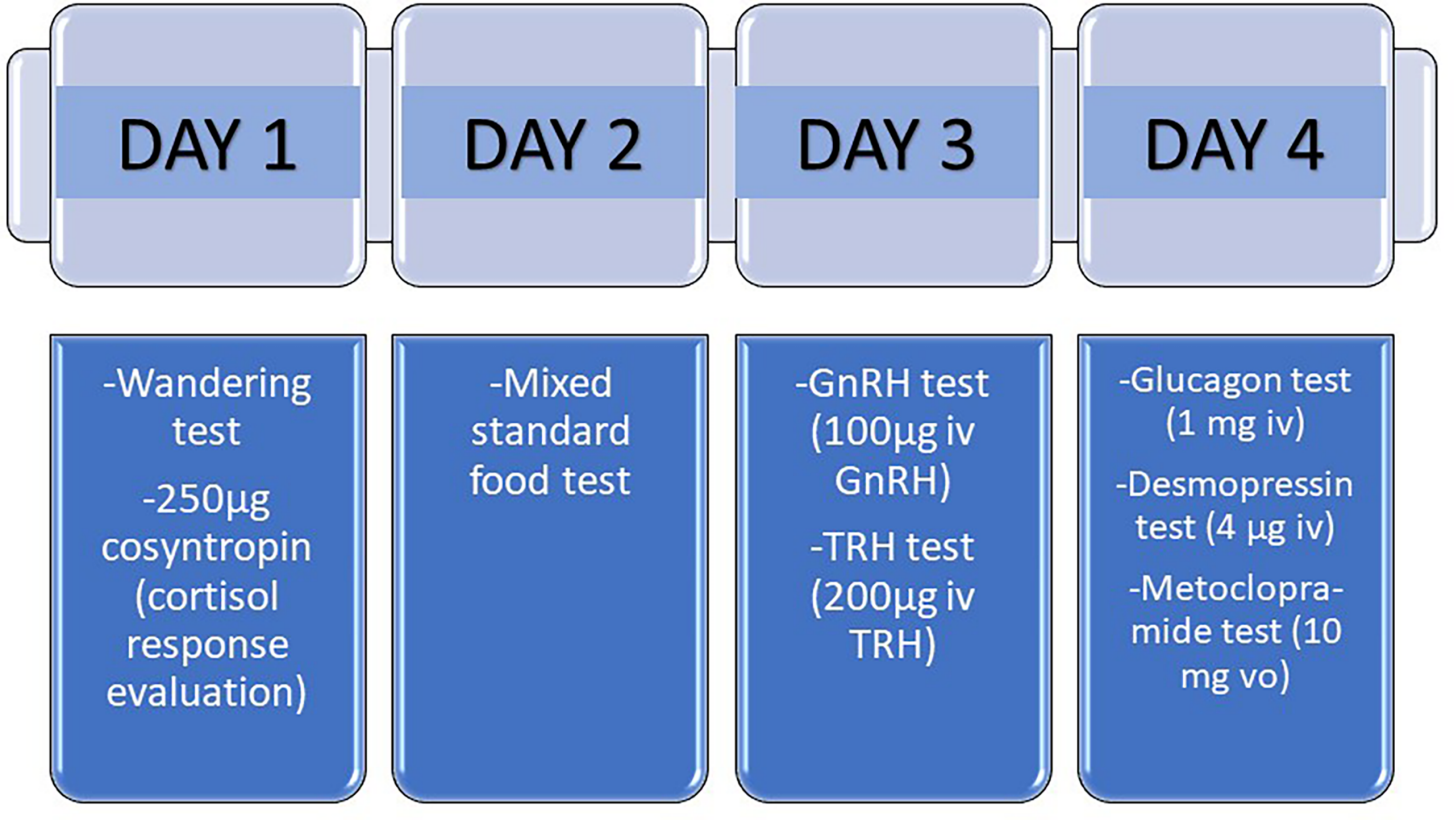
Protocol for the study of aberrant receptors in patients with adrenal Cushing´s syndrome. The study of aberrant receptors may be carried out in four consecutive or not-consecutive days. A change of < 25% of the cortisol level is considered as no response; a change between 25-49% as a partial response and a change ≥50% as a complete positive response to the test. Mixed standard food test consists in the administration of a mixed standard meal (450 calories, 46% of carbohydrates, 32% of lipids and 22% of proteins) and the measured of serum cortisol after 60 minutes of the ingestion and then every hour until complete 180 minutes. In the wandering test, the patient must walk for 2 hours, and then serum cortisol is measured every 30 minutes until complete 240 minutes.

There are different protocols to assess the presence of aberrant receptors, and within each protocol several variants. Generally, the study is carried out in four consecutive or not-consecutive days (35) **(** **Figure 3** **).** The parameter used to evaluate the response to the tests is the dynamics of cortisol after the administration of different stimuli. A change of < 25% of the cortisol level is considered as no response; a change between 25-49% as a partial response and a change ≥50% as a complete positive response to the test. However, there are some authors do not believe that the study of the aberrant receptors is of particular use today in clinical practice with respect to treatment. These authors argue that more than one aberrant receptor is identified in each patient, rendering it difficult to select a specific receptor to be blocked (10) and that the use of aberrant receptor antagonists does not usually allow long-term control of hypercortisolism. Moreover, they are expensive and time-consuming tests (43). Nevertheless, the evaluation of the presence of aberrant receptors offers the possibility of using specific drugs to block the identified aberrant hormone receptors. The option of performing an abbreviated protocol can be considered, performing only those tests for which there is an option of blocking treatment. In this sense, the ones with the highest performance would be to check the response to GIP with the mixed meal test, to beta adrenergics with the glucagon test and to LH/hCG with the GnRH test (**Figure 3**). This reduced protocol may be performed in just two days. Mixed standard meal test consists in the administration of a mixed standard meal (450 calories, 46% of carbohydrates, 32% of lipids and 22% of proteins) and the measured of serum cortisol after 60 minutes of the ingestion and then every hour until complete 180 minutes. In the glucagon and GnRH test, 1 mg of intravenous glucagon and 100µg of intravenous GnRH are administered, respectively, and then serum cortisol is measured every 30 minutes until complete 120 minutes.

### 2.8 Treatment of BMACD with overt Cushing´s syndrome

#### 2.8.1 Surgical treatment

In cases of overt CS, active treatment is necessary. Regarding surgical treatment, bilateral adrenalectomy is the classic treatment, but it involves a high risk of complications and life-long treatment with gluco- and mineralocorticoids. On the other hand, it has the advantage that there is no risk of recurrence if a complete resection is performed (17). Due to these drawbacks, the current trend is to perform a unilateral adrenalectomy with the resection of the largest adrenal gland or the one with the highest uptake of iodo-cholesterol. This approach avoids adrenal insufficiency in a high percentage of cases, and generally when it occurs it is transitory, and the risk of complications is lower (51). Unilateral adrenalectomy leads to control of hypercortisolism in up to 97% of the cases according to some series, although hypercortisolism recurs in 10-15% of patients, and even several years after the first surgery (52). However, in a recent series of 39 patients with BMACD, long-term hypercortisolism control rates (median of 50 months) were only of 32% and a hypercortisolism-related mortality rate of 15% was reported (53).

More recently, newer surgical approaches have been proposed to try to avoid the risks associated with bilateral adrenalectomy as well as minimize the risk of hypercortisolism recurrence that unilateral adrenalectomy entails. In a recent prospective study (54), a total adrenalectomy of the larger adrenal gland with a partial adrenalectomy of the contralateral gland were performed. This study included 17 patients with BMACD followed by a median of 41 months. With this approach, hypercortisolism was controlled in 95% of the cases, recovery of the pituitary-adrenal axis was achieved in 71% of cases, and there was only one recurrence of hypercortisolism after 30 months of follow-up.

#### 2.8.2 Medical treatment

The presence of aberrant receptors may offer the possibility to propose specific medical therapies. In case of response to GIP, octreotide or pasireotide can be used, although the improvement is usually transitory, and surgery ends up being necessary in most cases (55, 56). If there is a response to beta adrenergics or LH/hCG, propanolol (β adrenergics) or GnRH agonist/leuprolide acetate (LH/hCG) could be used, with cases of long-term hypercortisolism response described (57, 58).

In patients without aberrant receptors with a contraindication to surgery, or in order to achieve hypercortisolism control before the surgical intervention, steroidogenesis inhibitors such as ketoconazole or metopirone could be used (59). Cases of improvements in the glycemic control and in the body weight have also been reported with the use of mifepristone (60). Another glucocorticoid receptor antagonist, relacorilant, is under development for patients with adenomas or hyperplasia with associated hypercortisolism (https://clinicaltrials.gov/ct2/show/NCT04308590). Another phase I clinical trial investigates the usefulness of 11β-hydroxysteroid dehydrogenase type 1 inhibitors in patients with adrenal CS or ACS. To date, only 16 patients have been included, but the study described a significant improvement in plasma glucose levels, a reduction in the body mass index and the percentage of fat, and an increase in the percentage of muscle mass (61).

### 2.9 Treatment of BMACD with ACS

The treatment decision can be more complex in those patients with BMACD and ACS. We have the same treatment options than in overt CS, but also the option of follow-up without specific treatment for hypercortisolism.

If adrenalectomy is performed, it is recommended to be unilateral, resecting the largest gland (6, 9). Its indication should be considered in patients with ACS with potentially associated ACS comorbidities (especially type 2 diabetes mellitus, arterial hypertension and/or osteoporosis), particularly if they are poorly controlled and if the patient is young (<50-60 years-old) (47). Regarding adrenalectomy in ACS, the Bancos et al. meta-analysis (62) should be highlighted in this regard. This study focuses on comparing conservative vs. surgical management of ACS. It includes a total of 26 studies, 584 patients with ACS and 457 patients with non-functioning adrenal incidentalomas. As a main result, they found that compared to conservative treatment, patients with ACS who underwent an adrenalectomy, experienced a significant improvement in arterial hypertension (RR 11, 95% CI: 4.3-27.8) and type 2 diabetes mellitus (RR 3.9, 95% CI: 1.5-9.9). In this study, the effects of adrenalectomy in bone health were not analyzed. In fact, there is not a specific study focus on bone health evaluation and evolution after surgery in patients with BMACD and ACS. Nevertheless, a higher prevalence of vertebral fractures has been described in patients with impaired cortisol suppression (47). Moreover, in unilateral adrenal incidentalomas with ACS, adrenalectomy resulted in a reduction of 30% in fracture risk after recovery from cortisol excess (63). In terms of medical treatment, we have the same options as in overt CS, but experience with steroidogenesis inhibitors is low, and regarding aberrant receptor blocker treatment, the main issue is that it could be difficult to assess the response to treatment in some patients as UFC and midnight cortisol may be normal in patients with ACS (7). In a recent case report, ketoconazole resulted in a significant improvement in blood pressure in a patient with BMACD and ACS (59). In other study, metyrapone restore the physiological rhythm of cortisol in patients with ACS (64).

### 2.10 Follow-up in patients with BMACD and ACS

In patients diagnosed with BMACD, there is no specific recommendation for hormonal and imaging follow-up. Thus, the European Endocrine Society guidelines for the management of adrenal incidentalomas should be followed (49). Regarding radiological re-evaluation, although the guidelines recommend repeating the imaging tests in lesions larger than 4 cm, until now BMACD is an invariably benign condition, so it would not be necessary to repeat the radiological study if the diagnosis of BMACD is clear. Regarding hormonal follow-up, it would be the same as in patients with ACS from other causes, this being an annual control with DST and the assessment of the comorbidities potentially related to ACS, which are the ones that can mark the indication for surgery in these patients (62). Moreover, determinations of UFC, ACTH, DHEA-S should be considered.

## 3 Micronodular bilateral adrenal cortical disease (miBACD)

### 3.1 Definition

miBACD is characterized by micronodules (<1 cm) in both adrenal glands. Two types of miBACD are differentiated: i) PPNAD, generally associated with Carney syndrome, related to mutations in the alpha subunit of protein kinase A, and ii) i-MAD, which in a significant proportion of cases is also related to mutations in protein kinase A, but does not present with other clinical manifestations in addition to the adrenal hyperplasia (6). In total, three types of miBACD are distinguished: a) PPNAD as part of Carney complex (c-PPNAD), b) PPNAD not associated with Carney complex or isolated PPNAD (i-PPNAD), and c) i-MAD which does not have the characteristic pigmentation of PPNAD and is not associated with CNC (**Table 3**).

**Table 3:** Subtypes of bilateral micronodular adrenal cortical disease (65).

Subtype	Age of presentation (years)	Associated hereditary syndrome	Genetic defect
c-PPNAD	20-30	Carney complex (AD)	PRKAR1A, PDE8B
i-PPNAD	20-30	Isolated (AD)	PRKAR1A, PDE11A
i-MAD	30-40	Isolated (AD)	PRKAR1A, PDE11A, PDE8B

AD, autosomal dominant; c-PPNAD, pigmented nodular adrenocortical disease associated to Carney complex; i-PPNAD, isolated pigmented nodular adrenocortical disease associated; i-MAD, isolated micronodular adrenocortical disease.

PPNAD, the most common presentation of miBACD, is characterized by slightly enlarged or even normal adrenal glands containing multiple pigmented (lipofuscin) cortical micronodules usually smaller than 4 mm and surrounded by cortical internodal atrophy. In contrast, in the i-MAD form there is no nodular pigmentation or internodal atrophy (65, 66)

### 3.2 Pathophysiology

A disruption of PRKAR1A has been identified in PPNAD as one of the main pathophysiology mechanisms implied in the disease. PRKAR1A inactivation leads to the activation of both the Wnt/b-catenin pathway and the mTOR pathway, which impairs cell cycle and cell migration (67). Finally, pigmentation of adrenal nodules is due to the impairment of autophagy, that leads to the accumulation of lipofuscin at the nodules (68) (**Figure 1**).

### 3.3 Genetic

Regarding its genetics, PPNAD is generally transmitted as an autosomal dominant disease, being 70-80% of the cases related to Carney syndrome (2p16 and 17q22-24) (69). In patients with PPNAD and Carney syndrome, 65–82% had germline PRKAR1A mutations (70). These cases generally present with a more severe phenotype. Some genotype-phenotype correlation for PRKAR1A mutations have been previously described, for example certain tumors being more frequent with specific mutations in Carney syndrome (69). Germline mutations in phosphodiesterase isoform 11A (PDE11A) (2q31.2) (23) and isoform 8B (PDE8B) (5q13) (71) have been described in few cases of i-MAD (**Table 3** **).** Moreover, somatic beta-catenin gene (CTNNB1) mutations were found in larger adrenocortical nodules of 11% of patients with PPNAD in one series (63).

### 3.4 Epidemiology

PPNAD is hereditary in most cases, associated with Carney syndrome (c-PPNAD). In only 12% of patients, PPNAD appears to be isolated (i-PPNAD). More than 70% of cases occur in women, with age-related penetrance being higher in women than in men (70). Peak of age for hyperplasia is usually between 20-30 years, although there are cases reported in childhood (72). On the other hand, 60-70% of Carney syndrome patients have PPNAD (69). i-MAD is usually diagnosed between 30-40 years of age. i-MAD can be sporadic or familial (**Table 3**).

### 3.5 Clinical presentation and biochemical diagnosis

Patients may present with ACS or with overt CS, with sudden or insidious onset. It is one of the forms of CS that manifest early, in 50% it appears between 20-30 years of age, but there are even cases described with only 15 months of age in patients with Carney syndrome. In global terms, PPNAD represents the youngest etiology of primary adrenal CS. In PPNAD, cases of cyclic CS or even spontaneous resolution of CS have been reported (65). Cases of atypical presentation have also been described, such as some CS that appear during pregnancy, which could be related to cortisol stimulation by the high levels of estrogens (73)

As a characteristic feature, these patients present a paradoxical increase in plasma or urinary cortisol after the administration of high doses of dexamethasone, in approximately 70% of cases. In the Stratakis et al. study (74), a 6-day Liddle test was conducted in 21 patients with PPNAD: after 2 days of baseline measurement of urinary steroid excretion, dexamethasone, 0.5 mg, was given orally every 6 hours for 2 days starting at 6:00 a.m.; the dosage of dexamethasone was then increased to 2 mg every 6 hours for the last 2 days of the test. They found that a 100% increase in the UFC value on day 6 is highly specific for PPNAD (74). Other more recent study has evaluated the response of UFC after a low-dose dexamethasone suppression test (0.5 mg of dexamethasone every 6 h for 2 days) and high-dose dexamethasone suppression test (2 mg of dexamethasone every 6 h for 2 days) in patients with PPNAD and BMACD (75). They found a significant increase of UFC after the low- and high-doses tests in patients with PPNAD (paired t-test, P = 0.007 and P =0.001), while it remained unchanged in the BMACD group. In these patients, ACTH levels are completely suppressed, even with normal UFC.

Only 30% of children have growth retardation, which could be related to cyclical cases of CS or the presence of GH hypersecretion, which is common in patients with Carney syndrome (76). Another curious finding in this form of CS is the higher risk of osteoporotic fractures than in other forms of CS, which could be related to a longer duration of CS, but the cause remains unknown (77). Moreover, PRKAR1A gene mutation leads to an abnormal differentiation of osteoblasts.

On the other hand, the Carney syndrome is a rare multiple neoplasia syndrome, characterized by the presence of pigmented lesions in the skin and/or mucous membranes, myxomas in the heart and in other tissues, and benign and malignant tumors in the thyroid, testicles, pancreas, and pituitary gland, among other manifestations. About 70% of cases are familial with autosomal dominant transmission, and the remaining cases are currently considered sporadic (72, 78). PPNAD is the most frequent endocrine manifestation of this syndrome. For the diagnosis of Carney complex syndrome, it is required that there are at least two major manifestations or one major and one supplementary manifestation (70) ***(*** **Table 4** ***).***

**Table 4:** Criteria for Carney Complex (72).

Major diagnosis criteria	Supplemental criteria
1. Spotty skin pigmentation with a typical distribution (lips, conjunctiva and inner or outer canthi, vaginal and penile mucosa) 2. Myxoma (cutaneous and mucosal) 3. Cardiac myxoma 4. Breast myxomatosis or fat-suppressed MRI findings suggestive of this diagnosis 5. PPNAD or paradoxical positive response of urinary glucocorticosteroids to dexamethasone administration during Liddle’s test 6. Acromegaly due to GH-producing adenoma 7. LCCSCT or characteristic calcification on testicular ultrasonography 8. Thyroid carcinoma or multiple, hypoechoic nodules on thyroid ultrasonography, in a young patient 9. Psammomatous melanotic schwannoma 10. Blue nevus, epithelioid blue nevus (multiple) 11. Breast ductal adenoma (multiple) 12. Osteochondromyxoma	1. Affected first-degree relative 2. Inactivating mutation of the PRKAR1A gene

LCCSCT, large cell calcifying Sertoli cell tumor; MRI, magnetic resonance imaging; PPNAD, pigmented nodular adrenocortical disease.

To make a diagnosis of Carney Syndrome, a patient must either: 1) exhibit two of the major manifestations of the disease or 2) exhibit one of these manifestations and meet one of the supplemental criteria.

### 3.6 Radiological diagnosis

CT can show adrenal glands of normal characteristics in up to 30% of cases of miBACD. In these cases, it is important to perform a CT scan with slices of 3 mm or less, in which micronodules appearing as hypodense spots scattered throughout the surface of the adrenal glands. In addition, macronodules have been described in less than 5% of cases, being more frequent in the elderly (65). [6β-131I]iodomethyl-19-norcholesterol] [NP-59] scintigraphy may be of useful in the diagnosis of miBACD, especially in those cases with normal appearance on CT, since NP-59 scintigraphy always shows a bilateral adrenal uptake in miBACD, confirming the bilateral nature of the disease. However, asymmetrical scintigraphic uptake can be observed in patients with macronodules (79). Despite the low experience with adrenal venous sampling in miBACD, it could be useful in patients with normal CT and/or [NP-59] scintigraphy (65).

Although it is a benign condition, 2 cases of adrenocortical carcinoma and 1 case of pheochromocytoma have been described in patients with PPNAD (65).

### 3.7 Treatment

Before considering surgery, it is important to rule out Carney complex, and especially the presence of cardiac myxomas to avoid complications during surgery. In patients with Carney syndrome, echocardiograms should be performed annually if there is no history of cardiac myxoma, or twice a year in patients with previously excised myxomas (80)

In patients with PPNAD, bilateral adrenalectomy is usually the first line of treatment. It leads to the complete remission of the CS in almost every case. However, it causes permanent adrenal insufficiency and requires lifelong use of hormone replacement (glucocorticoid and mineralocorticoid), altering the patient´s quality of life and increasing the risk of complications (65). For these reasons, unilateral adrenalectomy has been proposed as a novel technique, with the main objective of avoiding adrenal insufficiency. It could be assessed in cases of mild hypercortisolism and asymmetric disease (51). CS remission rates are approximately 65% in some series, although there is little experience with this surgical technique for this disease (51).

Medical treatment with adrenal enzyme inhibitors (ketoconazole or metyrapone, mitotane) would be primarily reserved as preoperative treatment in patients with overt CS.

## 4 Conclusions

The main causes of primary bilateral adrenal cortical disease that may lead to hypercortisolism are BMACD and miBACD. They are characterized by the size of the nodules found in the hyperplasia larger or smaller than 1 cm respectively. BMACD may be associated with overt CS or ACS. Up to 55% of cases of BMACD with overt CS are associated with germline mutations in ARMC5. In addition, more than 75% of BMACD cases had an activation of the cAMP/PKA pathway through stimulation by ligands other than ACTH of aberrantly expressed G protein-coupled receptors. Surgical treatment should be based on the resection of the largest adrenal gland, since it leads to hypercortisolism remission in up to 95% of the cases. Medical treatment focused on the blockade of aberrant receptors may lead to hypercortisolism control in some cases. miBACD is a rare cause of adrenal CS, representing one of the main causes of CS at a young age. It may be divided into at least two main different conditions: PPNAD and i-MAD. Its more common presentation is familial PPNAD, as part of Carney syndrome. A paradoxical increase in 24h-urinary free cortisol in the high-doses dexamethasone suppression test is a quite specific data of PPNAD. Bilateral adrenalectomy is generally the treatment of choice in patients with overt CS and miBACD, but unilateral adrenalectomy may be considered in cases with asymmetric disease and mild hypercortisolism

## Author contributions

Writing—original draft preparation: MA-C. Figure creation: MA-C and MM. Writing—review and editing: MA-C and MM. All authors contributed to the article and approved the submitted version.

## Funding

IIS LA PRINCESA – Instituto de Investigación del Hospital de la Princesa. Diego de León street, N° 62 PC 28006, Madrid, Spain. This work was supported by the following grants: Proyectos de Investigacion en Salud (FIS) PI16-02091 and PI19-00584 (funded by Instituto de Salud Carlos III), TIRONET2-CM, B2017/BMD-3724 (funded by Comunidad de Madrid), and cofinanced by FEDER funds to MM.

## Conflict of interest

The authors declare that the research was conducted in the absence of any commercial or financial relationships that could be construed as a potential conflict of interest.

## Publisher’s note

All claims expressed in this article are solely those of the authors and do not necessarily represent those of their affiliated organizations, or those of the publisher, the editors and the reviewers. Any product that may be evaluated in this article, or claim that may be made by its manufacturer, is not guaranteed or endorsed by the publisher.
